# Clinical analysis of 2860 cases of diabetes in pregnancy: a single-center retrospective study

**DOI:** 10.1186/s12884-022-04712-0

**Published:** 2022-05-18

**Authors:** Jia Chen, Zhenyu Wang, Weizhen Wu, Haixia Chen, Caijuan Zhong, Lixuan Liang, Yingtao Li

**Affiliations:** 1 Department of Obstetrics, The Third Affiliated Hospital of Guangzhou Medical University, Guangzhou, 510150 China; 2 Department of Obstetrics, Foshan Women and Children hospital, Foshan, 528000 China; 3 Department of Obstetrics and Gynecology, Sun Yat-sen Memorial Hospital of Sun Yat-sen University, Guangzhou, 510120 China; 4 Department of Obstetrics, Guangdong Women and Children Hospital, Guangzhou, 510010 China; 5 Guangzhou Medical Centre for Critical Pregnant Women, Guangzhou, 510150 China; 6 Guangdong Provincial Key Laboratory of Major Obstetric Diseases, Guangzhou, 510150 China

**Keywords:** Pregnancy, Diabetes, Clinical characteristic, Insulin, Pregnancy outcome

## Abstract

**Background:**

To investigate the epidemiological, clinical characteristics and outcomes of diabetes in pregnancy (DIP).

**Methods:**

This single-center, retrospective study included 16,974 pregnant women hospitalized during 2018–2019. Among them, 2860 DIP patients were grouped according to diabetes type, glycemic status, and insulin use. Multivariate logistic regression analysis was conducted.

**Results:**

The incidence of DIP [17.10%; pregestational diabetes mellitus (PGDM), 2.00% (type I, 0.08%; type 2, 1.92%); gestational diabetes mellitus (GDM), 14.85% (GDM A1, 13.58%; GDM A2, 1.27%)] increased annually. Premature birth, congenital anomalies, large for gestational age (LGA), neonatal asphyxia, neonatal intensive care unit transfer, hypertension, and puerperal infection were more common in DIP than in healthy pregnancies. The most common comorbidities/complications were hypertension, thyroid dysfunction, cervical incompetence, intrahepatic cholestasis, premature membrane rupture, oligo/polyhydramnios, and fetal distress. GDM incidence at ages ≥35 and ≥ 45 years was 1.91 and 3.26 times that at age < 35 years, respectively. If only women with high-risk factors were screened, 34.8% GDM cases would be missed. The proportion of insulin use was 14.06% (PGDM, 55%; GDM, 8.53%). Mean gestational age at peak insulin dose in DIP was 32.87 ± 5.46 weeks. Peak insulin doses in PGDM and GDM were 3.67 and 2 times the initial doses, respectively. The risks of LGA, premature birth, cesarean section, and neonatal hypoglycemia in PGDM were 1.845, 1.533, 1.797, and 1.368 times of those in GDM, respectively. The risks of premature birth and neonatal hypoglycemia in women with poor glycemic control were 1.504 and 1.558 times of those in women with good control, respectively.

**Conclusions:**

The incidence of adverse outcomes in DIP is high.

## Background

Diabetes is one of the most important diseases affecting human health. In recent years, with the liberalization of the two-child policy in China, there has been an increase in the number of older women and obese women who become pregnant. Furthermore, owing to the transformation of gestational diabetes screening strategies, the incidence of diabetes in pregnancy (DIP) has increased significantly [1, 2]. DIP includes pregestational diabetes mellitus (PGDM) and gestational diabetes mellitus (GDM). PGDM mainly includes type 1 diabetes mellitus (T1DM) and type 2 diabetes mellitus (T2DM). GDM refers to diabetes that first occurs and is diagnosed during pregnancy, and it is one of the most common obstetric complications. GDM can be categorized into GDM A1 and GDM A2.

According to the data released by the International Diabetes Federation in 2019, the incidence of DIP was 15.8%, with GDM accounting for 83.6% of cases [3]. In 2018, the New Zealand Ministry of Health reported that the incidence of PGDM was 1.12%, that of T1DM was 0.36%, and that of T2DM was 0.75% [4]. According to recent reports, the incidence of GDM in China is as high as 14.8% [5].

GDM usually manifests as a hyperglycemic state caused by relatively insufficient insulin secretion due to a gradual increase in insulin resistance in the middle and late stages of pregnancy, which is similar to T2DM. The mechanism underlying the development of GDM can be described as follows: fasting blood glucose levels decrease with the progression of pregnancy; however, due to impaired insulin-mediated glucose utilization, inhibition of endogenous glucose sources, and insufficient first-phase increase in insulin secretion, the postprandial increase in blood glucose levels is abnormal, with large fluctuations and a long duration. For women with PGDM, the existing dysfunction of the pancreatic islets is superimposed on the physiological changes in glucose metabolism in pregnancy; furthermore, the levels of insulin antagonists in the body increase with gestational age. Thus, the amount of insulin required to maintain normal glucose metabolism increases [6].

At present, there is no global consensus on the diagnostic methods and standards for GDM. A glucose tolerance test in the first trimester for women at a high risk for GDM and routine screening for all pregnant women at 24–28 weeks of gestation can help reduce the incidence of adverse pregnancy outcomes [7]. Studies [8, 9] have found that high blood glucose levels can have adverse effects on the mother and fetus. Women with DIP who are in a hyperglycemic state for prolonged periods are prone to systemic arteriolar vascular disease, which leads to poor intrusion of the villous trophoblast cells into the spiral artery of the uterus, causing implantation failure and increasing the risk of abortion and stillbirth [10]. Systemic arteriolar vascular disease can also increase the risk of maternal hypertension during pregnancy. In addition, DIP may increase the risk of obesity and T2DM in the offspring. Nielsen et al. [11] found that increased glycosylated hemoglobin (HbA1c) is related to pregnancy outcomes. For every 1% increase in the HbA1c level, the risk of adverse pregnancy outcomes increases by 3.8 to 7.3%. Davidson et al. [12] found that for every 0.5% decrease in the HbA1c level, the risks of fetal and fetal heart malformations were reduced by 1 and 11%, respectively. In pregnant women with diabetes, maternal hyperglycemia can lead to fetal hyperglycemia. Excessive glucose supply is considered key in DIP-related macrosomia [13]. When the newborn is delivered, the hyperglycemic environment disappears; however, the insulin level in the newborn remains high, which leads to the occurrence of neonatal hypoglycemia [14].

The treatment of DIP mainly includes diet, exercise, self-monitoring of blood glucose, health education, and drug treatment. In addition, close maternal and fetal monitoring should be performed. At present, insulin is the first-line medication for the treatment of hyperglycemia in women with DIP. Most studies have shown that 10 to 36% of GDM patients require insulin [15, 16]. In the case of T1DM patients, the maximum insulin dose required during pregnancy is at least twice that required before pregnancy; in addition, pregnant women with T2DM often require insulin treatment or increasing insulin doses during 28–32 weeks of gestation, which is a period of rapid fetal development [17]. Good glycemic control is key to good maternal and fetal prognoses; however, there is no global consensus on the goal of blood glucose control, and many factors can affect the prognosis of women with DIP.

In view of this, we conducted a retrospective study to investigate the epidemiological characteristics of DIP in the recent 5 years, the proportion of adverse pregnancies, clinical characteristics and pregnancy outcome in our hospital in Guangzhou, which is a treatment center for severely ill pregnant women, following the official launch of the second-child policy in China on January 1, 2016. We analyzed the clinical characteristics of DIP patients and the high-risk factors for GDM, explored the regularity of insulin use in DIP patients, and examined the adverse pregnancy outcomes of DIP and its influencing factors. The aim of this study was to provide some guidance for standardize the clinical management of DIP in the future.

## Methods

### Research subjects

This retrospective study reviewed the data of 16,974 pregnant women admitted to the Third Affiliated Hospital of Guangzhou Medical University between January 1st, 2018 and December 31st, 2019. Of these, 2860 women met the selection criteria were included in this study. The inclusion criteria were pregnant women who (a) met the diagnostic criteria for DIP, (b) delivered their baby in our hospital, and (c) had complete data. Patients with incomplete data were excluded. All methods were carried out in accordance with relevant guidelines and regulations. The Ethics Committee approval number is GD2019–033. Informed consent was obtained on-line from all participants.

### Data collection

**Fig. 1:**
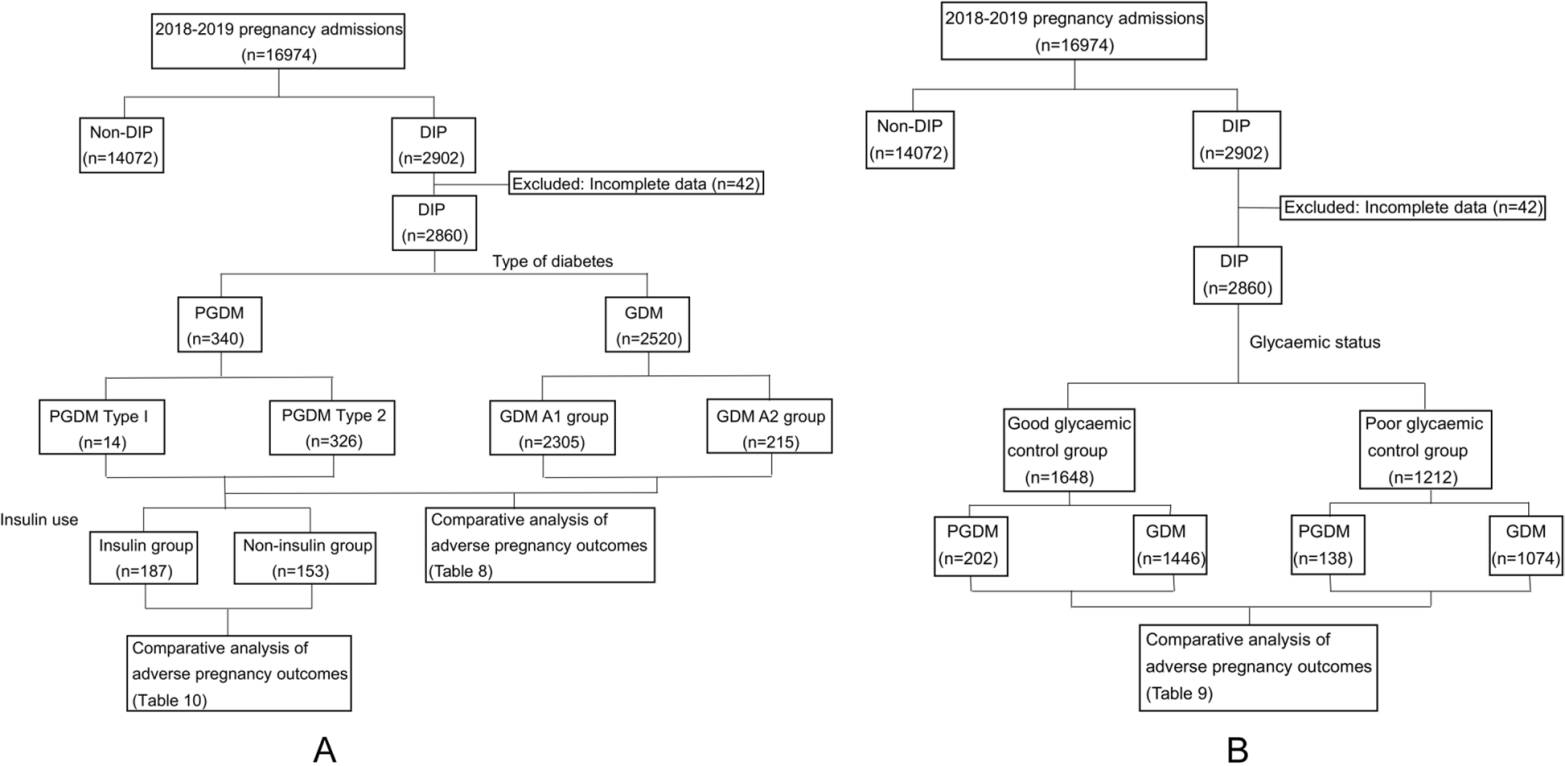
Flowchart of study. Comparisons of pregnancy outcomes in women with DIP, grouped according to the (**A**) type of diabetes (overall cohort) and insulin use (PGDM group) and (**B**) glycemic status. DIP, diabetes in pregnancy; PGDM, pregestational diabetes mellitus; GDM, gestational diabetes mellitus

The medical record system of the hospital was screened to collect patient data during hospitalization. The information gathered included age, birth history, method of conception, number of pregnancies, height, pre-pregnancy weight, body mass index (BMI), total pregnancy weight increment, gestational age, type of delivery, maternal comorbidities and complications, and neonatal conditions. A flow chart of the study is shown in Fig. 1A and B.

### Diagnostic criteria for DIP

#### Diagnostic criteria for PGDM

Patients who met any of the two following criteria were diagnosed with PGDM: (1) Patients who had been diagnosed with diabetes before pregnancy. (2) Pregnant women who had not undergone blood glucose testing before pregnancy, particularly, those with high-risk factors for diabetes, had to undergo a check for diabetes during their first prenatal examination. These women were diagnosed with PGDM if they met any of the following criteria: fasting plasma glucose (FPG) ≥ 7.0 mmol/L; blood glucose level 2 h after a 75-g oral glucose tolerance test (OGTT) ≥ 11.1 mmol/L, accompanied by typical hyperglycemic symptoms or hyperglycemic crisis; random blood glucose ≥11.1 mmol/L; and HbA1c ≥ 6.5% [18].

#### Diagnostic criteria for GDM

(1) All pregnant women who had not yet been diagnosed with GDM or PGDM underwent the 75-g OGTT at their first visit at 24–28 weeks or after 28 weeks of gestation. The diagnostic criteria for GDM with the 75-g OGTT were as follows: fasting blood glucose ≥5.1 mmol/L, 1-h blood glucose ≥10.0 mmol/L, and 2-h blood glucose ≥8.5 mmol/L. GDM could be diagnosed if any of the above was met. (2) Pregnant women with high-risk factors for GDM or those who lived in areas with insufficient medical resources had to check their FPG at 24–28 weeks of pregnancy. An FPG level ≥ 5.1 mmol/L could be used to directly diagnose GDM without the 75-g OGTT [19].

All GDM diagnoses were made at 24–28 weeks of gestation. If the results of an OGTT in early pregnancy were lower than the WHO standard, but met the WHO/International Association of Diabetes and Pregnancy Study Groups GDM criteria, the patient was considered to be at a high risk for GDM and offered health education.

The White classification defines GDM A1 as fasting blood glucose < 5.3 mmol/L and 2-h postprandial blood glucose < 6.7 mmol/L after dietary control, and GDM A2 as fasting blood glucose ≥5.3 mmol/L and 2-h postprandial blood glucose ≥6.7 mmol/L after dietary control [20].

### Incidence of adverse pregnancy

#### Definitions

##### Maternal complications

Hypertensive disorders of pregnancy (HDPs): gestational hypertension, chronic hypertension with preeclampsia, eclampsia, and pregnancy with chronic hypertension.

Intrahepatic cholestasis of pregnancy (ICP): skin pruritus with fasting serum total bile acid level ≥ 10 μmol/L.

Abnormal thyroid function during pregnancy: hyperthyroidism or hypothyroidism.

Polyhydramnios: amniotic fluid volume > 2000 mL (indicated on ultrasonography by ≥8 cm vertical depth of the largest dark area of amniotic fluid or an amniotic fluid index ≥25 cm).

Oligohydramnios: amniotic fluid volume < 300 mL in the third trimester (indicated on ultrasonography by ≤2 cm vertical depth of the largest dark zone of amniotic fluid or an amniotic fluid index ≤5 cm).

Abortion: termination of pregnancy when the embryo or fetus is not viable. In China, a termination is termed abortion, if the pregnancy has not reached 28 weeks, and the fetal weight < 1000 g.

Premature delivery: delivery at > 28 weeks of gestation but < 37 weeks of gestation.

Congenital anomalies: any structural or functional metabolism abnormality during the development of the embryo or fetus detected by B-ultrasound monitoring during pregnancy. Congenital anomalies diagnosed after birth were not included since long-term follow-up of the newborns was not conducted in this study.

Stillbirth: fetal death in utero or during delivery after 20 weeks of gestation.

Postpartum hemorrhage (PPH): blood loss > 500 mL after vaginal birth or > 1000 mL after cesarean delivery within 24 h after the fetus is delivered.

##### Neonatal conditions

Small for gestational age (SGA): newborns or fetuses whose birth weight is lower than the 10th percentile of weight for gestational age.

Large for gestational age (LGA): newborns or fetuses whose birth weight is higher than the 90th percentile for gestational age.

Macrosomia: birth weight > 4000 g.

Neonatal hypoglycemia: According to the diagnostic criteria in our country is blood glucose level < 2.2 mmol/L [7].

Hyperbilirubinemia: total bilirubin level exceeding the 95th percentile.

Neonatal asphyxia: Apgar score ≤ 7 at 1 min or 5 min.

### Nutrition management

The daily energy factor required for women with DIP was selected according to their pre-pregnancy BMI and labor intensity. Their daily calorie requirements were calculated according to the difference between the pre-pregnancy weight and the weight gain rate during pregnancy [7, 21] by using the following formula: daily calorie requirement = standard weight × energy factor (additional 200 kcal/d in the middle and late stages of pregnancy), in which standard weight = (height - 70) × 60%. The recommended daily calorie intake in the first and third trimesters were ≥ 1500 kcal and ≥ 1800 kcal, respectively. The daily proportions of the three major nutrients were 50–60% of carbohydrates, 25–30% of fat, and 15–20% of protein. The patients were recommended to choose foods with a low glycemic index (GI), consume 25–30 g/d of dietary fiber, and limit table salt to < 6 g/d.

### Exercise therapy

The women were recommended to exercise for 15–20 min at 30 min after each meal [22]. The exercise intensity could be medium-intensity aerobic exercise or resistance exercise. There were two ways to judge the suitable exercise intensity for the pregnant women: (1) after exercising for not less than 15 min, the heartbeat speeds up, but the subject does not feel fatigued; and (2) after exercising, the maximum oxygen consumption is 40–60%, there is moderate sweating, and the muscles have a slight feeling of soreness [22].

### Target blood glucose value

For GDM patients, the target blood glucose levels before meals and at 2 h after meals were ≤ 5.3 and ≤ 6.7 mmol/L, respectively, and the target HbAlc level was < 5.5% [21]. For PGDM patients, the target pre-meal and fasting blood glucose levels were 3.3–5.6 mmol/L; the target peak blood glucose level at 2 h after meals was 5.6–7.1 mmol/L; and the target HbAlc level was < 6.0%. Fasting, pre-meal, or postprandial blood glucose levels ≥20% of the above standards were considered to indicate poor glycemic control. GDM and PGDM patients with HbAlc levels of ≥5.5% and ≥ 6.0%, respectively, were also considered to have poor glycemic control [21].

Indications for insulin use: GDM patients with blood glucose level > 5.3 mmol/L before meal and > 6.7 mmol/L at 2 h after meal, and PGDM patients with blood glucose level > 5.6 mmol/L before meal and > 7.1 mmol/L at 2 h after meal.

### Online-to-offline management mode for DIP

The hospital has established a multidisciplinary DIP cooperation mode and an innovation office for gestational diabetes, which has transformed conventional offline medical treatment by enabling face-to-face individualized obstetric health care and practical training sessions that are conducted for two and a half days a week. In addition, WeChat public accounts were used for health education. WeChat groups have graphics, text, and audio messaging, instant online interactions, convenient sharing, and privacy.

### Grouping

The patients were divided into groups according to (1) type of diabetes: GDM A1, GDM A2, and PGDM subgroups; (2) glycemic status: glycemic and poor glycemic control subgroups; and (3) insulin use in the PGDM group: insulin and no-insulin subgroups.

### Data analysis

SPSS software, version 26.0 (IBM Corp., Armonk, NY, USA) was used for data analysis. Count data were presented as percentages or frequencies. Comparisons between groups were performed using the Fisher exact test or χ^2^ test. Measurement data that were normally distributed were represented using mean ± standard deviation. Data with non-normal distribution were represented using medians and interquartile ranges. If two sets of samples conformed to a normal distribution, the independent-samples *t*-test was used for comparison. For samples with non-normal distribution, the non-parametric Wilcoxon rank-sum test was used. Logistic regression analysis was used for multivariate analysis. *P* < 0.05 indicated that the difference was statistically significant.

## Results

### Epidemiological characteristics of DIP

**Fig. 2:**
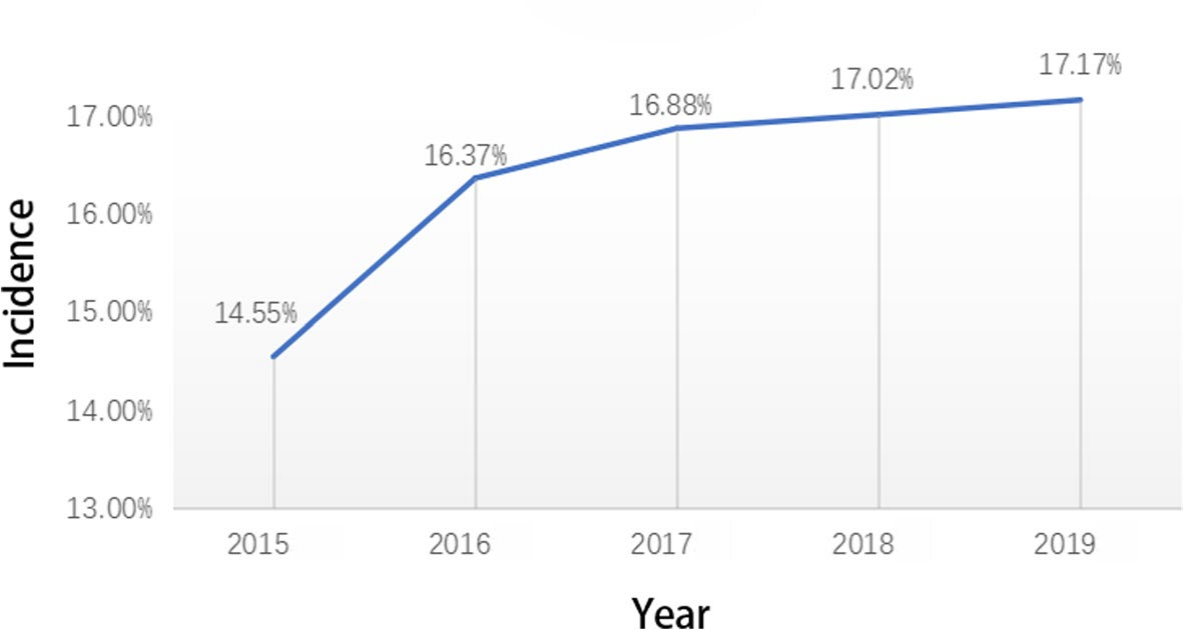
Annual incidence of diabetes in pregnancy in the recent 5 years

We calculated the annual incidence of DIP in our hospital in the recent 5 years. On January 1, 2016, the second-child policy in China was officially launched. The number of older and obese pregnant women has gradually increased, and the incidence of DIP has increased significantly. The incidence of DIP in our hospital in 2015 was 14.55% (1001/6878), while in 2016, it was 16.37% (1322/8077), which is a significant increase from 2015. The incidence of DIP in 2017, 2018, and 2019 was 16.88% (1392/8244), 17.02% (1413/8300), and 17.17% (1489/8674), respectively, which demonstrates a trend of annual increase (Fig. 2).

### Incidence of adverse pregnancy outcomes in hospitalized pregnant women with and without DIP during 2018–2019

From January 1, 2018 to December 31, 2019, the total number of hospitalizations for pregnancy was 16,974 (DIP, 2902 cases; non-DIP, 14072 cases). The incidence of DIP was 17.10% (2902/16974). The incidence of PGDM was 2.00% (340/16974; type I, 0.08% [14/16974]; type 2, 1.92% [326/16974]), and the incidence of GDM was 14.85% (2520/16974; GDM A1, 13.58% [2305/16974]; GDM A2, 1.27% [215/16974]).

**Fig. 3:**
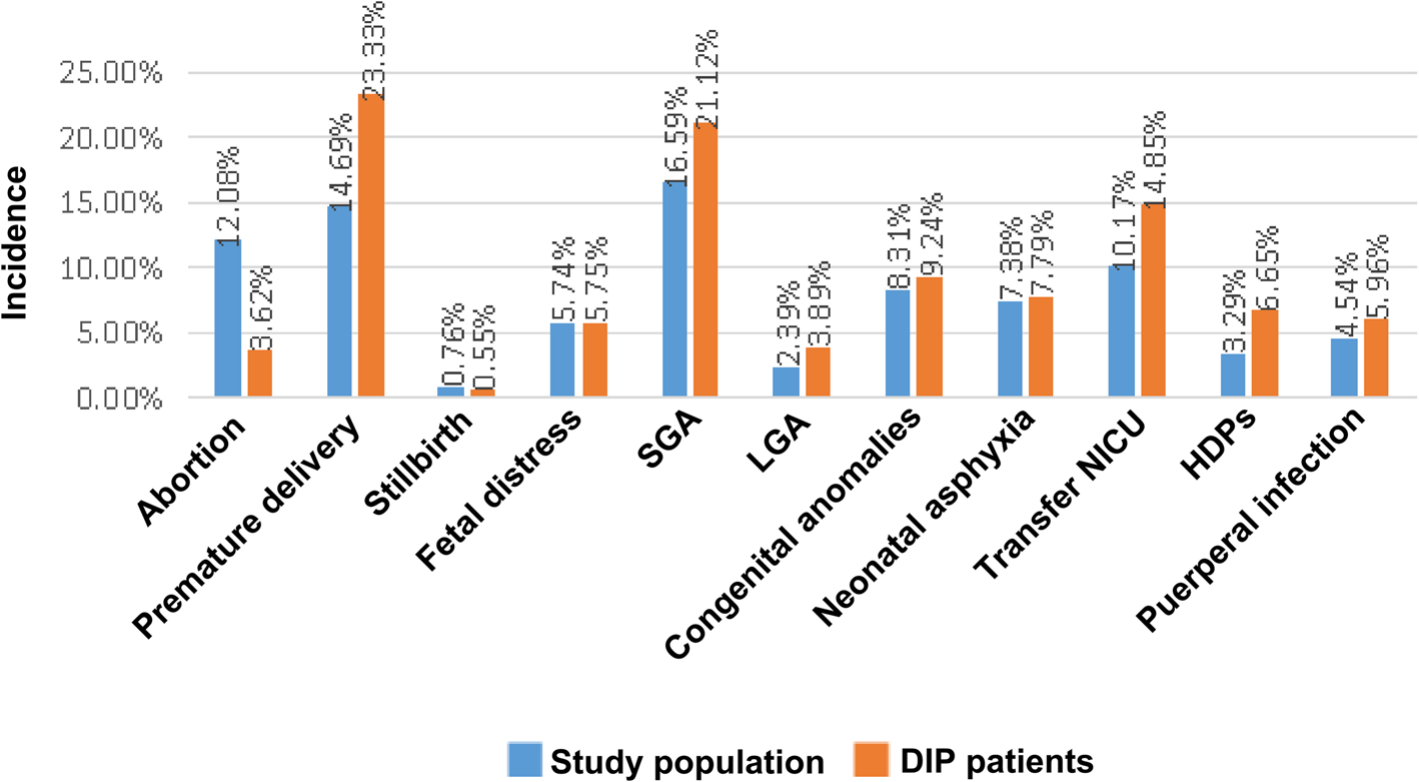
Comparison of the incidence of adverse pregnancy outcomes in the study population and hospitalized DIP patients. DIP, diabetes in pregnancy; SGA, small for gestational age; LGA, large for gestational age; NICU, neonatal intensive care unit; HDPs, hypertensive disorders of pregnancy

Among the non-DIP population, the incidence rates of various outcomes were as follows: premature delivery, 12.91% (1816/14072); stillbirth, 0.80% (113/14072); fetal distress, 5.73% (807/14072); SGA, 15.64% (2201/14072); LGA, 2.08% (292/14072); congenital anomalies, 8.12% (1142/14072); neonatal asphyxia, 7.29% (1026/14072); transfer to the neonatal intensive care unit (NICU), 9.20% (1295/14072); HDPs, 2.59% (365/14072); and puerperal infection, 4.24% (597/14072). The incidence of premature delivery, congenital anomalies, large for gestational age (LGA), neonatal asphyxia, transfer to NICU, hypertensive disorders of pregnancy (HDPs), and puerperal infection was higher in hospitalized pregnant women with DIP than in those without DIP (Fig. 3).

### General condition, comorbidities, and complications of hospitalized patients with DIP

The mean age of the DIP patients was 33.56 ± 4.86 years, of whom 41.40% (1184/2860) were patients with advanced maternal age (age ≥ 35), 33.88% (969/2860) were overweight or obese, and 22.41% (641/2860) had undergone in vitro fertilization and embryo transfer (IVF-ET). There were 2575 cases of singleton pregnancies, 282 cases of twin pregnancies, and 3 cases of triplet pregnancies. The mean total weight gain during pregnancy was 12.05 ± 4.76 kg, and the mean hospital stay was 5.5 ± 3.7 days (Table 1). The incidence of comorbidities and complications among hospitalized DIP patients is shown in Table 2.

**Table 1:** General condition of women hospitalized due to DIP

Variable	Mean ± SD/Median	Range
Age (yrs)	33.56 ± 4.86	18–51
BMI (kg/m^2^)	22.84 ± 3.59	12.5–38.94
Gravida	2 (1, 3)	1–11
Parity	1 (0, 1)	0–6
Number of abortions (n)	0 (0, 1)	0–6
Number of cesarean sections (n)	0 (0, 1)	0–3
Number of examinations (n)	9 (7, 10)	0–22
Total weight gain during pregnancy (kg)	12.05 ± 4.76	−3 to 43
Length of stay (d)	5.5 ± 3.7	1–42

*DIP* Diabetes in pregnancy, *BMI* Body mass index

**Table 2:** Comorbidities and complications among women hospitalized due to DIP

Variable	No. of cases	Incidence (%)
HDP	193	6.75
Pregnancy complicated with thyroid dysfunction	144	5.03
ICP	39	1.36
Cervical incompetence	103	3.60
Polyhydramnios	30	1.05
Oligohydramnios	175	6.12
PROM	538	18.81
Fetal distress	167	5.84
Puerperal infection	173	6.05

*DIP* Diabetes in pregnancy, *HDP* Hypertensive disorders of pregnancy (HDP), *ICP* intrahepatic cholestasis of pregnancy, *PROM* Premature rupture of membranes

In all, 27 of 2860 (0.94%) DIP patients required transfer to the ICU, including 6 (22.22%) patients with PGDM and 21 (77.78%) patients with GDM. Among the PGDM patients, ICU transfer was necessitated by diabetic ketoacidosis, hemorrhagic shock, severe preeclampsia, hypoxemia, severe acute pancreatitis, and multiple organ dysfunction syndromes (1 patient each, accounting for 16.67%). The main causes of ICU transfer among the GDM patients were hemorrhagic shock and pregnancy complicated with heart disease, each accounted for 23.81% of ICU transfers (Table 3).

**Table 3:** Etiological analysis of DIP patients transferred to the ICU

Reason for transfer	PGDM (***N*** = 6)		GDM (***N*** = 21)	
	Number	%	Number	%
Hemorrhagic shock	1	16.67	5	23.81
Severe preeclampsia	1	16.67	1	4.76
Diabetic ketoacidosis	1	16.67	0	0
Heart disease in pregnancy	0	0	5	23.81
Septic shock	0	0	1	4.76
Acute fatty liver	0	0	4	19.05
Hypoxemia	1	16.67	2	9.52
Pregnancy with hyperlipidemia	0		1	4.76
Severe acute pancreatitis	1	16.67		0
Intracranial hemorrhage	0	0	1	4.76
Multiple organ dysfunction syndrome	1	16.67	0	0
Pheochromocytoma	0	0	1	4.76

*DIP* Diabetes in pregnancy, *ICU* Intensive care unit, *PGDM* Pregestational diabetes mellitus, *GDM* Gestational diabetes mellitus

### Perinatal insulin usage in DIP

Insulin use was recorded in 14.06% (402/2860) of all women with DIP, 55% (187/340) of women with PGDM, and 8.53% (215/2520) of women with GDM. The mean gestational age at the time of the peak insulin dose was 32.87 ± 5.46 weeks among women with DIP, and this parameter did not differ between the PGDM and GDM groups (*P* > 0.05; Table 4). The mean gestational age at the start of insulin use was significantly lower, and the mean initial and maximum insulin doses were significantly higher in the PGDM group than in the GDM group (*P* < 0.05). The highest insulin dose in the PGDM and GDM groups was 3.67 times (1.9 times, 7.42 times) and 2 times (1 time, 4.4 times) the initial dose, respectively. The difference between the two groups was statistically significant (*P* < 0.05). In the PGDM and GDM groups, the insulin dosage was decreased in the third trimester in 95 (50.80%) and 102 (47.44%) women, respectively (*P* > 0.05). The median dosage used (and quartiles) in the PGDM and GDM groups before delivery was 37 U (16 U, 60 U) and 9.5 U (2.0 U, 22.25 U), respectively (*P* < 0.05).

**Table 4:** Characteristics of insulin use among women with DIP

Variable	Group		***t*** value	***P*** value
	GDM + insulin	PGDM + insulin		
Gestational age at start of insulin use (wks)	29.29 ± 4.81	17.44 ± 11.03	13.58	< 0.05
Initial insulin dose (U)	7.80 ± 5.88	15.78 ± 13.90	−7.27	< 0.05
Gestational age at peak insulin dose (wks)	33.22 ± 3.91	32.48 ± 6.80	1.30	0.194
Maximum insulin dose (U)	22.07 ± 19.82	55.70 ± 34.60	−11.69	< 0.05

*DIP* Diabetes in pregnancy, *GDM* Gestational diabetes mellitus, *PGDM* Pregestational diabetes mellitus

Data are presented as mean ± standard deviation

Postpartum insulin use was required in 85 women with DIP, including 12 women with GDM A2, 14 women with type I diabetes, and 59 women with type 2 diabetes. Among the 12 women with GDM A2, 5 patients forgot the specific duration of postpartum insulin use, and 7 patients stopped using insulin immediately after discharge. All 14 women with type I diabetes continued using insulin beyond the postpartum period. Among the 59 women with type 2 diabetes, 16 patients forgot the specific duration of postpartum insulin use, and another 16 patients stopped using insulin immediately after discharge. Furthermore, 12 women continued using insulin beyond the postpartum period. The duration of postpartum insulin use among the remaining 15 women was as follows: 1 week, 1 woman; 1 month, 6 women; 42 days, 2 women; 3 months, 2 women; 6 months, 1 woman; 1 year, 2 women; and 2 years, 1 woman. The difference between the PGDM and GDM groups was statistically significant (*P* < 0.05). The mean postpartum insulin dosage in the PGDM and GDM groups was 32.07 and 26.20% of the antepartum dosage, respectively.

### High-risk factors for GDM

**Fig. 4:**
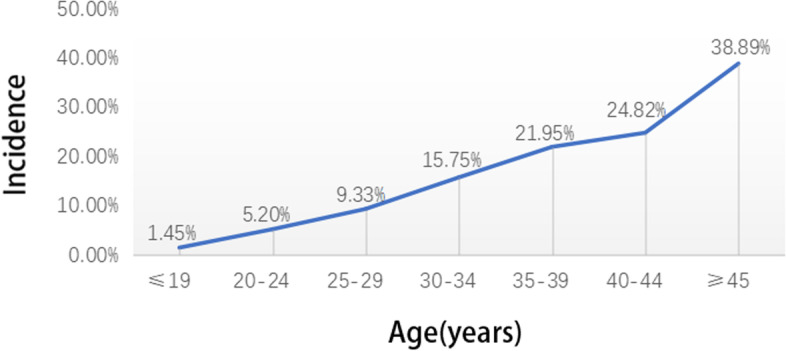
Relationship between age and incidence of gestational diabetes mellitus

Table 5 shows patients with GDM and all pregnant women in this study stratified by age group. The relationship between age and the incidence of GDM is illustrated in Fig. 4. The incidence of GDM increased with age in a linear upward trend. The incidence of GDM at the ages of ≥35 and ≥ 45 years was 1.91 and 3.26 times that at the age of < 35 years. The incidence of GDM at age ≥ 45 years was as high as 38.89%. The main high-risk factors for GDM are shown in Table 6. A comprehensive analysis of 7 high-risk factors found that GDM with at least one high-risk factor accounted for 65.20% (1643/2520) of all cases of GDM. The mean gestational age at the time of GDM diagnosis significantly differed between women with high-risk factors and those without high-risk factors (25.41 ± 2.92 weeks vs. 25.97 ± 2.51 weeks, *P* < 0.05).

**Table 5:** Age distribution of women with GDM and pregnant women without diabetes

Age (yrs)	Women with GDM (n)	Pregnant women without diabetes (n)
≥45	35	90
40–44	235	947
35–39	738	3362
30–34	967	6140
25–29	481	5154
20–24	63	1212
≤19	1	69

*GDM* Gestational diabetes mellitus

**Table 6:** High-risk factors for GDM

Risk factor	n (%)
Overweight	614 (24.37)
Obesity	191 (7.58)
History of diabetes in first-degree relatives	188 (7.46)
History of fetal macrosomia	32 (1.27)
History of GDM	91 (3.61)
History of HDPs	249 (9.88)
Polycystic ovary syndrome	126 (5.00)

*GDM* Gestational diabetes mellitus, *HDPs* Hypertensive disorders of pregnancy

### Between-group comparisons of pregnancy outcomes in women with DIP

#### Grouping according to the type of diabetes

Of the 2520 women with GDM, 91.47% (2305/2520) had GDM A1, and 8.53% (215/2520) had GDM A2. Of the 340 women with PGDM, 4.12% (14/340) had type 1 diabetes, and 95.88% (326/340) had type 2 diabetes. A comparison of the incidence of pregnancy outcomes between these groups is presented in Table 7.

**Table 7:** Comparison of pregnancy outcomes between women with GDM A1, GDM A2, and PGDM

Variable	GDM A1 group (***N*** = 2305)	GDM A2 group (***N*** = 215)	PGDM group (***N*** = 340)	χ^**2**^ value	***P*** value
Premature delivery	515 (22.34%)^#^	55 (25.58%)^#^*	107 (31.47%)*	14.13	< 0.05
Stillbirth	14 (0.61%)	0	2 (0.59%)		0.787^a^
SGA	485 (21.04%)	52 (24.19%)	76 (22.35%)	1.35	0.509
LGA	83 (3.60%)^#^	6 (2.79%)^#^*	24 (7.06%)*	10.16	< 0.05
Congenital anomalies	212 (9.20%)	21 (9.77%)	35 (10.29%)	0.46	0.793
Neonatal asphyxia	175 (7.59%)	14 (6.51%)	37 (10.88%)	5.03	0.081
NICU transfer	317 (13.75%)^#^	33 (15.35%)^#^	81 (23.82%)*	23.49	< 0.05
Cesarean section	1186 (51.45%)^#^	126 (58.60%)^#^*	226 (66.47%)*	29.06	< 0.05
Assisted vaginal delivery	15 (0.65%)	2 (0.93%)	6 (1.76%)		0.076^a^
PPH	219 (9.50%)	17 (7.91%)	30 (8.82%)	0.67	0.706
Puerperal infection	134 (5.18%)	14 (6.51%)	25 (7.35%)	1.32	0.516
Maternal ICU transfer	21 (0.91%)	0 (0.00%)	6 (1.76%)		0.115^a^
Neonatal hypoglycemia	34 (1.48%)^#^	5 (2.33%)^#^*	12 (3.53%)*		< 0.05^a^
Neonatal hyperbilirubinemia	28 (1.21%)	1 (0.47%)	2 (0.59%)		0.536^a^

*GDM* Gestational diabetes mellitus, *PGDM* Pregestational diabetes mellitus, *SGA* small for gestational age, *LGA* Large for gestational age, *NICU* Neonatal intensive care unit, *PPH* Postpartum hemorrhage, *ICU* Intensive care unit

Note: Similar symbols between two groups indicate that the difference between the groups was not statistically significant; different symbols between two groups indicate statistically significant difference

^a^indicates the use of the Fisher exact test

#### Grouping according to the glycemic status

Of the total number of women with DIP, 57.62% (1648/2860) had good glycemic control [GDM, 57.38% (1446/2520); PGDM, 59.41% (202/340)], and 42.38% (1212/2860) had poor glycemic control [GDM, 42.62% (1074/2520); PGDM, 40.59% (138/340)]. The incidence of premature delivery, stillbirths, LGA, and neonatal hypoglycemia significantly differed between these two groups (*P* < 0.05; Table 8).

**Table 8:** Comparison of pregnancy outcomes between women with good and poor glycemic control

**Variable**	**No. of cases**	**Good glycemic control**			**Poor glycemic control**			**χ**^**2**^ **value**	***P*** **value**
		**GDM (*N*** **= 1446)**	**PGDM (*N*** **= 202)**	**Total (*N*** **= 1648)**	**GDM (*N*** **= 1074)**	**PGDM (*N*** **= 138)**	**Total (*N*** **= 1212)**		
Premature delivery	677	290 (20.06%)	53 (26.24%)	343 (20.81%)	295 (27.47%)	39 (28.26%)	334 (27.56%)	17.58	< 0.05
Stillbirth	16	5 (0.35%)	0 (0.00%)	5 (0.30%)	9 (0.84%)	2 (1.45%)	11 (0.91%)	4.58	< 0.05
SGA	613	292 (20.19%)	47 (23.27%)	339 (20.57%)	248 (23.09%)	26 (18.84%)	274 (22.61%)	1.72	0.190
LGA	113	36 (2.49%)	7 (3.47%)	43 (2.61%)	59 (5.49%)	11 (7.97%)	70 (5.78%)	18.45	< 0.05
Congenital anomalies	268	126 (8.71%)	15 (7.43%)	141 (8.56%)	113 (10.52%)	14 (10.14%)	127 (10.48%)	3.04	0.081
Neonatal asphyxia	226	108 (7.47%)	18 (8.91%)	126 (7.65%)	89 (8.29%)	11 (7.97%)	100 (8.25%)	0.35	0.553
NICU transfer	431	199 (13.76%)	43 (21.29%)	242 (14.68%)	158 (14.71%)	31 (22.46%)	189 (15.59%)	0.45	0.502
Cesarean section	1538	739 (51.11%)	120 (59.41%)	859 (52.12%)	593 (55.21%)	86 (62.32%)	679 (56.02%)	4.27	< 0.05
Assisted vaginal delivery	23	10 (0.69%)	1 (0.50%)	11 (0.67%)	9 (0.84%)	3 (2.17%)	12 (0.99%)	0.91	0.340
PPH	266	144 (9.96%)	18 (8.91%)	162 (9.83%)	92 (8.57%)	12 (8.70%)	104 (8.58%)	1.29	0.256
Puerperal infection	173	83 (5.74%)	18 (8.91%)	101 (6.13%)	61 (5.68%)	11 (7.97%)	72 (5.94%)	0.04	0.835
Maternal ICU transfer	27	12 (0.83%)	3 (1.49%)	15 (0.91%)	9 (0.84%)	3 (2.17%)	12 (0.99%)	0.05	0.827
Neonatal hypoglycemia	51	15 (1.04%)	3 (1.49%)	18 (1.09%)	26 (2.42%)	7 (5.07%)	33 (2.72%)	10.60	< 0.05
Neonatal hyperbilirubinemia	31	14 (0.97%)	2 (0.99%)	16 (0.97%)	15 (1.40%)	0 (0.00%)	15 (1.24%)	0.46	0.496
**Variable**		**Good glycemic control (*N*** **= 1648)**			**Poor glycemic control (*N*** **= 1212)**			**t value**	***P*** **value**
		**GDM (*N*** **= 1446)**	**PGDM (*N*** **= 202)**	**Mean (*N*** **= 1648)**	**GDM (*N*** **= 1446)**	**PGDM (*N*** **= 202)**	**Mean (*N*** **= 1648)**		
Gestational age at delivery (wks)		37.42 ± 3.05	36.80 ± 3.49	37.34 ± 3.11	37.08 ± 3.29	36.72 ± 3.36	37.04 ± 3.29	8.89	< 0.05
Volume of PPH (mL)		463.68 ± 331.38	472.55 ± 336.79	464.76 ± 331.95	465.86 ± 281.84	470.95 ± 257.14	466.44 ± 279.03	2.11	0.887

*GDM* Gestational diabetes mellitus, *PGDM* Pregestational diabetes mellitus, *SGA* small for gestational age, *LGA* Large for gestational age, *NICU* Neonatal intensive care unit, *PPH* Postpartum hemorrhage, *ICU* Intensive care unit

Data are presented as n (%) or mean ± standard deviation

The mean gestational age at delivery in the good and poor glycemic control groups were 37.34 ± 3.11 weeks and 37.04 ± 3.29 weeks, respectively; the difference was statistically significant (*P* < 0.05). The incidence of cesarean section was significantly lower in the good glycemic control group than in the poor glycemic control group (52.12% vs. 56.02%, *P* < 0.05; Table 8).

### Comparison of pregnancy outcomes in women with PGDM

#### Grouping according to insulin usage

In the PGDM group, 55% (187/340) of women were on insulin treatment. Women with PGDM who did not use insulin received combined treatment consisting of health education, diet modification, exercise, and blood glucose monitoring. The incidence of premature delivery and neonatal asphyxia significantly differed between PGDM patients who did and did not require insulin (*P* < 0.05). The mean gestational age at delivery in the insulin and non-insulin groups was 36.59 ± 3.31 and 35.64 ± 4.45 weeks, respectively; the difference was statistically significant (*P* < 0.05). The incidence of postpartum hemorrhage did not differ between the two groups (*P* > 0.05), but the mean volume of postpartum hemorrhage was significantly higher in the non-insulin group than in the insulin group (493.18 ± 295.45 mL vs. 432.93 ± 216.49 mL, *P* < 0.05; Table 9).

**Table 9:** Comparison of pregnancy outcomes between women with PGDM treated with and without insulin

Variable	No. of cases	Insulin group (***N*** = 187)	No-insulin group (***N*** = 153)	χ^**2**^ value	***P*** value
Premature delivery	107	49 (26.20%)	58 (37.91%)	5.35	< 0.05
Stillbirth	2	1 (0.53%)	1 (0.65%)		1.000^a^
SGA	76	36 (19.25%)	40 (26.14%)	2.30	0.129
LGA	24	17 (9.09%)	7 (4.58%)	2.62	0.106
Congenital anomalies	35	17 (9.09%)	18 (11.76%)	0.65	0.420
Neonatal asphyxia	37	13 (6.95%)	24 (15.69%)	6.62	< 0.05
NICU transfer	6	2 (1.70%)	4 (2.61%)		0.156^a^
Cesarean section	226	125 (66.84%)	101 (66.01%)	0.03	0.872
Assisted vaginal delivery	6	2 (1.07%)	4 (2.61%)		0.415^a^
PPH	30	12 (6.42%)	18 (11.76%)	2.99	0.084
Puerperal infection	25	11 (5.88%)	14 (9.15%)	1.32	0.251
Maternal ICU transfer	6	2 (1.07%)	4 (2.61%)		0.415^a^
Neonatal hypoglycemia	12	7 (3.74%)	5 (3.27%)	0.06	0.813
Neonatal hyperbilirubinemia	2	0	2 (1.31%)		0.202^a^

*PGDM* Pregestational diabetes mellitus, *SGA* Small for gestational age, *LGA* Large for gestational age, *NICU* Neonatal intensive care unit, *PPH* Postpartum hemorrhage, *ICU* Intensive care unit

^a^indicates the use of the Fisher exact test

### Multivariate analysis of adverse pregnancy outcomes in DIP

#### Premature delivery

Regression analysis showed that IVF-ET, HDPs, polyhydramnios, cervical insufficiency, premature rupture of membranes (PROM), PGDM, and poor glycemic control were factors affecting premature delivery (*P* < 0.05). IVF-ET, HDPs, and PROM increased the risk of premature delivery by 83.5, 58.4, and 98.9%, respectively. Polyhydramnios increased this risk by 1.236 times (adjusted odds ratio [aOR] = 2.236, 95% confidence interval [CI]: 1.053 to 4.749). PGDM was associated with 1.533 times (aOR = 1.533, 95% CI: 1.187 to 1.979) the risk of premature delivery than was GDM. Poor glycemic control was associated with 1.504 times (aOR = 1.504, 95% CI: 1.259 to 1.796) the risk of premature delivery than was good glycemic control (Table 10).

**Table 10:** Multivariate regression analysis of premature delivery in women with DIP

Variable	B	SE	WALS	df	***P***	aOR	95% CI
Age	0.004	0.009	0.144	1	0.704	1.004	0.985–1.022
IVF-ET	0.607	0.102	35.340	1	0.000	1.835	1.502–2.242
HDPs	0.460	0.167	7.611	1	0.006	1.584	1.142–2.196
Polyhydramnios	0.805	0.384	4.382	1	0.036	2.236	1.053–4.749
Cervical incompetence	0.730	0.212	11.866	1	0.001	2.076	1.370–3.146
PROM	0.687	0.107	41.305	1	0.000	1.989	1.613–2.452
PGDM	0.427	0.130	10.737	1	0.001	1.533	1.187–1.979
Poor glycemic control	0.408	0.091	20.267	1	0.000	1.504	1.259–1.796
Constant	−6.884	0.713	93.112	1	0.000	0.001	

*DIP* Diabetes in pregnancy, *SE* Standard error, *WALS* Weighted-average least squares, *df* Degrees of freedom, *aOR* Adjusted odds ratio, *CI* Confidence interval, *IVF-ET* In vitro fertilization-embryo transfer, *HDPs* Hypertensive disorders of pregnancy, *PROM* premature rupture of membranes, *PGDM* Pregestational diabetes mellitus

#### LGA

Regression analysis showed that PGDM, poor glycemic control, and total weight gain during pregnancy were factors influencing LGA (*P* < 0.05). PGDM was associated with 1.845 times the risk of LGA than was GDM (aOR = 1.845, 95% CI: 1.051 to 3.240). Poor glycemic control was associated with 2.479 times the risk of LGA than was good glycemic control (aOR = 2.479, 95% CI: 1.606 to 3.825). Every 1 kg of total weight gain during pregnancy increased the risk of LGA by 5.5% (aOR = 1.055, 95% CI: 1.015 to 1.096; Table 11).

**Table 11:** Multivariate regression analysis of LGA in the DIP group

Variable	B	SE	WALS	df	***P***	aOR	95% CI
Polyhydramnios	1.249	0.636	3.851	1	0.050	3.486	1.002–12.134
PGDM	0.613	0.287	4.548	1	0.033	1.845	1.051–3.240
Poor glycemic control	0.908	0.221	16.825	1	0.000	2.479	1.606–3.825
Total weight gain during pregnancy	0.053	0.019	7.509	1	0.006	1.055	1.015–1.096
Constant	−8.514	1.190	51.211	1	0.000	0.000	

*LGA* Large for gestational age, *DIP* Diabetes in pregnancy, *SE* Standard error, *WALS* weighted-average least squares; *df* Degrees of freedom, *aOR* Adjusted odds ratio, *CI* confidence interval, *PGDM* Pregestational diabetes mellitus

Adjusted variables consisted of age, polyhydramnios, PGDM, poor glycemic control, total weight gain during pregnancy, and no insulin use

#### Neonatal hypoglycemia

Regression analysis showed that poor glycemic control and PGDM were factors influencing neonatal hypoglycemia (*P* < 0.05). Poor glycemic control was associated with 2.558 times the risk of neonatal hypoglycemia than was good glycemic control (OR = 2.558, 95% CI: 1.432 to 4.568). PGDM was associated with 2.368 times the risk of neonatal hypoglycemia than was GDM (OR = 2.368, 95% CI: 1.225 to 4.579; Table 12).

**Table 12:** Multivariate regression analysis of cesarean section in the DIP population

Variable	B	SE	WALS	df	***P***	aOR	95% CI
Age	0.035	0.009	14.157	1	0.000	1.036	1.017–1.055
IVF-ET	1.089	0.104	109.450	1	0.000	2.973	2.424–3.646
PGDM	0.586	0.137	18.224	1	0.000	1.797	1.373–2.351
LGA	0.565	0.228	6.136	1	0.013	1.760	1.125–2.753
Scarred uterus	2.543	0.125	413.407	1	0.000	12.714	9.951–16.246
Oligohydramnios	0.641	0.180	12.750	1	0.000	1.899	1.335–2.700
^a^Constant	−8.559	0.623	188.821	1	0.000	0.000	
Poor glycemic control	0.939	0.296	10.074	1	0.002	2.558	1.432–4.568
PGDM	0.862	0.336	6.564	1	0.010	2.368	1.225–4.579
^b^Control	−8.183	1.208	45.919	1	0.000	0.000	

*DIP* Diabetes in pregnancy, *SE* Standard error, *WALS* Weighted-average least squares, *df* Degrees of freedom, *aOR* Adjusted odds ratio, *CI* Confidence interval, *IVF-ET* In vitro fertilization-embryo transfer, *PGDM* Pregestational diabetes mellitus, *LGA* Large for gestational age

^a^Adjusted variables consisted of age, IVF-ET, PGDM, LGA, scarred uterus, oligohydramnios, insulin use, and poor glycemic control

^b^Adjusted variables consisted of poor glycemic control, PGDM, LGA, and no insulin use

#### Cesarean section

Regression analysis showed that age, IVF-ET, PGDM, LGA, scarred uterus, and oligohydramnios were factors influencing of cesarean section (*P* < 0.05). For every 1 year increase in age, the risk of cesarean section increased by 3.6% (aOR = 1.036, 95% CI: 1.017 to 1.055). IVF-ET increased the risk of cesarean section by 1.973 times (aOR = 2.973, 95% CI: 2.424 to 3.646). PGDM was associated with 1.797 times the risk of cesarean section than was GDM (aOR = 1.797, 95% CI: 2.424 to 3.646). The risk of cesarean section among women with a scarred uterus increased by 11.714 times compared to women with a healthy uterus (aOR = 12.714, 95% CI: 9.951 to 16.246). LGA and oligohydramnios increased the risk of cesarean section by 76 and 89.9%, respectively (Table 12).

## Discussion

### Epidemiological characteristics of DIP

The present study showed that the incidence of DIP is higher in China than in other countries, while the incidence of GDM is similar [3, 5, 6]. The high incidence of DIP may be related to the establishment of the diabetes innovation center in our hospital, which receives many patients who are referred from other hospitals.

### Incidence of adverse pregnancy outcomes in hospitalized pregnant women with and without DIP during 2018–2019

Poor glycemic control in DIP results in poor maternal and fetal prognoses. Previous studies found that the incidence rates of abortion and stillbirth in DIP are 10–17% [23] and 5.9% [24], respectively, while the incidence rates of premature delivery in PGDM and GDM are 14.24 and 8.98%, respectively [25]. The aORs of PGDM and GDM for fetal malformations were 2.44 and 1.28, respectively [26]. The incidence rates of macrosomia in T1DM and T2DM were 51 and 38%, respectively [27]. The results of our study suggested that DIP has a higher incidence of adverse pregnancy outcomes, and thus, it warrants greater clinician attention.

### General condition, comorbidities, and complications of hospitalized patients with DIP

HDPs are one of the most common complications of DIP. Women with DIP are 2–4 times more likely to develop HDPs than women without DIP [7, 28]. When diabetes is accompanied by microvascular disease, particularly renal microvascular disease, the incidence of HDPs and preeclampsia can be as high as above 50% [7]. Women with HDPs often have abnormal glucose metabolism, and these two conditions affect each other. In this study, HDPs were the main comorbidity of DIP, accounting for 6.75% of cases. PROM was the main complication, accounting for 18.81% of cases. This finding may be explained as follows: abnormal glucose metabolism can easily alter the vaginal flora, which reduces the local tension of the fetal membranes and results in PROM. Therefore, glycemic control in pregnancy may help reduce the occurrence of PROM. Polyhydramnios may be related to fetal hyperglycemia, hyperosmolar diuresis, and increased fetal urine excretion. Bicocca et al. [29] found that the incidence of polyhydramnios in DIP was 10.5%. However, the incidence of polyhydramnios in this study was low (1.05%), and the incidence of puerperal infection (6.05%) was consistent with that in the normal population (6%) [7]. These findings may be attributable to the establishment of the innovative O2O office for GDM by our team, which helped to achieve good glycemic control in DIP, and thereby reduce the incidence of polyhydramnios and puerperal infections. For women who had a normal OGTT in the second trimester but are found to have polyhydramnios in the third trimester, the OGTT may be repeated to detect latent GDM in a timely manner. For women with third-trimester polyhydramnios who have already been diagnosed with DIP, the diabetes diet and exercise treatment plan should be adjusted as soon as possible.

In recent years, DIP associated with cervical incompetence has garnered increasing attention from obstetricians. The incidence of cervical incompetence in this study was 3.60%, which is higher than the rates of 0.1–1.0% reported in the literature [30]. This may be related to the fact that our hospital is a treatment center for severely ill pregnant women in Guangdong Province, the establishment of an innovative office for the management of cervical incompetence, and the referral of many patients with cervical incompetence from other hospitals. Cervical incompetence is an important factor for abortion and premature delivery in the second trimester. Treatment with progesterone, or cervical cerclage, and relative bed rest are the methods used to manage this condition.

### High-risk factors for GDM

Studies have suggested that the high-risk factors for GDM include race, advanced age, overweight or obesity, history of diabetes among first-degree relatives, and previous history of macrosomia or GDM, HDPs, and polycystic ovary syndrome [31]. Asians are a high-risk group for GDM. According to the previous reports, among the high-risk factors for GDM, age ≥ 32 years and overweight accounted for 27.9 and 28.3%, respectively [32]. The risk of GDM increases by 1.883 times when the maternal age is ≥36 years [33]. The present study showed that advanced age is the most common risk factor for GDM, accounting for 40% of cases, followed by overweight, which accounted for 24.37% of cases. The incidence of GDM increases with age. Owing to changes in our lifestyle, the number of overweight or obese women has been increasing yearly, which may be one of the reasons for the increase in the incidence of GDM. Giving birth at a suitable age, if possible, can reduce the risk of GDM. Women who are overweight or obese before pregnancy can undertake lifestyle interventions for proper weight control or weight loss to help reduce their risk of GDM and adverse pregnancy complications. Zhang et al. [34] found that the incidence of GDM among women undergoing assisted reproductive technology (ART) treatment was 1.9 times that among women who conceived naturally. The use of progesterone in early pregnancy may have an impact on glucose metabolism and increase the incidence of GDM. The present study found that IVF-ET accounted for 22.18% of GDM cases, and is very likely to be a high-risk factor for GDM. In addition, women undergoing IVF-ET have an increased rate of multiple pregnancies, which is also a high-risk factor for GDM. In this study, 65.20% of women diagnosed with GDM had at least one high-risk factor. Consistent with this, O’Sullivan et al. [35] found that if only women with high-risk factors for GDM were screened using OGTTs, the diagnosis rate of GDM would be 62%. The mean gestational age at the time of GDM diagnosis was lower in women with high-risk factors than in women without high-risk factors. Thus, early screening of those with high-risk factors for GDM can not only increase the diagnosis rate of GDM but also lead to the early detection of GDM. This is useful because early intervention can effectively reduce the occurrence of adverse pregnancy outcomes in such patients. Nevertheless, if only those with high-risk factors for GDM are screened, the missed diagnosis rate will be 34.8%. Therefore, while early screening of women with high-risk factors is recommended, women without high-risk factors should be routinely screened during the period of rapid fetal growth period at 24–28 weeks.

### Perinatal insulin usage in DIP

After standardized diet and exercise treatment, if the blood glucose still does not reach the target, medication should be promptly added. At present, insulin is the first-line treatment for hyperglycemia due to DIP. It has been reported that 24.1–33.7% of GDM patients require insulin [36, 37]. Among PGDM patients, the maximum insulin dose to treat T1DM during pregnancy is at least twice as high as that required before pregnancy, while T2DM often necessitates additional insulin treatment or a rapid increase in insulin dose during 28–32 gestational weeks, which is a period of rapid fetal development [17]. Padmanabhan et al. [38] found that in the third trimester, the required insulin dose increased by 22.9% in T1DM and by 44% in T2DM; furthermore, patients with T1DM had a slight decrease in insulin dose before delivery, while those with T2DM did not. Roeder et al. [39] found that the insulin dose immediately after delivery in T1DM patients should be 30–35% less than the pre-pregnancy dose. If the pre-pregnancy insulin dose is unknown, the insulin dose should be reduced to 50% of the pre-delivery dose and adjusted according to the blood glucose level.

The present study found that the incidence rates of insulin use were 14.06% in DIP patients, 55% in PGDM patients, and 8.53% in GDM patients, which are lower than the rates reported in the literature [36, 37]. This finding may be related to the implementation of the O2O management mode and the standardized implementation of diet and exercise in our hospital. The mean gestational age at which insulin treatment was initiated was lower in the PGDM group than in the GDM group, and the mean initial and maximum insulin doses were higher in PGDM group than in the GDM group. These findings may be related to the presence of islet dysfunction in women with PGDM and the aggravation of insulin resistance during pregnancy. The mean gestational age at the time of the peak insulin dose in women with DIP was 32 weeks, and this parameter did not significantly differ between the PGDM and GDM groups, indicating that in both groups, insulin resistance was most obvious during the period of rapid fetal development, and it was necessary to adjust the dose of insulin in a timely manner to maintain a stable blood glucose level.

In the third trimester, the required insulin dose decreased in 50.80% of women with PGDM and 47.44% of women with GDM, with no significant between-group difference. This is inconsistent with the results reported by Padmanabhan et al. [38], and may be related to the failure to detect the type of PGDM in this study and to distinguish between the doses used in the third trimester of pregnancy and the pre-delivery period. Our results suggested that GDM is also associated with the phenomenon of physiological insulin reduction in the third trimester.

In the present study, postpartum insulin use was required in 21.14% of women with DIP, 39.04% of women with PGDM, and 5.58% of women with GDM. The mean postpartum insulin dose was 32.07 and 26.20% of the antepartum dose in the PGDM and GDM groups, respectively, which is consistent with the literature [7, 39]. With the delivery of the placenta, insulin resistance is significantly reduced, and the insulin concentration is significantly increased. Therefore, the insulin dose should be carefully adjusted in the postpartum period, particularly for breastfeeding patients. It is recommended that the initial postpartum dose be one-third of the pre-delivery or pre-pregnancy dose to avoid the occurrence of hypoglycemia.

### Between-group comparisons of pregnancy outcomes in women with DIP

#### Grouping according to type of diabetes

The PGDM group had a significantly higher incidence of premature delivery, LGA, and neonatal hypoglycemia than the GDM A1 group, and a significantly higher incidence of NICU transfers than the GDM A1 and GDM A2 groups.

#### Grouping according to glycemic status

The poor glycemic control group had a significantly higher incidence of premature delivery, stillbirth, LGA, neonatal hypoglycemia, cesarean delivery, and lower gestational age at delivery than the good glycemic control group.

#### Grouping according to insulin usage

The no-insulin group had a significantly higher incidence of premature delivery and neonatal asphyxia than the insulin group.

### Multivariate analysis of adverse pregnancy outcomes in DIP

#### Abortion

The incidence of abortion in the DIP group was 3.62%, which is lower than that reported in the literature [7]. This may be due to the fact that our hospital is a specialized treatment center for severely ill pregnant women, and few patients who require abortion seek admission into our hospital. To reduce the incidence of abortion in DIP, we recommend the following measures: (1) in the case of women with PGDM, the blood glucose level should be controlled within the normal range before pregnancy, (2) the HbA1c level should be < 6.5%, and (3) multidisciplinary assessment should be used to determine whether these women can safely become pregnant.

#### Premature delivery

The incidence of premature delivery in DIP was 10–25%. The risk of polyhydramnios in women with DIP was 10 times that in women without DIP. The higher the blood glucose level, the more common is polyhydramnios, which can lead to premature delivery [7]. Lin et al. [40] found that among women with GDM, the incidence of premature delivery was significantly higher in those with poor glycemic control than in those with good glycemic control.

The present study found that the incidence of premature delivery in DIP was 23.33%, which is consistent with that reported in the literature [7]. The incidence of premature delivery in the PGDM group was significantly higher than that in the GDM A1 and A2 groups, and the incidence in the GDM A2 group was significantly higher than that in the GDM A1 group. Compared with GDM, PGDM was associated with 1.533 times the risk of premature delivery, which is consistent with the risk reported in the literature [15].

During pregnancy, women with DIP require more frequent blood glucose monitoring, good control of the blood glucose level within the standard range, and timely insulin treatment when necessary to reduce the incidence of premature delivery.

#### Stillbirth

Reports on the incidence of stillbirth vary globally, with the reported rates in developed and developing countries being 3.1 and 30%, respectively [41, 42]. In China, the incidence of stillbirth is 8.8% [43]. If DIP is not controlled, hyperglycemia and diabetic ketoacidosis can occur, which can lead to stillbirth. A large study [24] analyzed the data of 10,733,983 newborns in the United States from 1995 to 1997. The results showed that the risk of stillbirth was higher in women with DIP than in women without DIP (5.9% vs. 4.0%). Tennant et al. [44] found that the risk of stillbirth was increased by 4 times in women with PGDM compared to women without DIP. In the third trimester, HbA1c > 5.8% and lack of antenatal folic acid supplementation were the only variables that were significantly associated with stillbirth. HbA1c > 6.6% was independently associated with the risk of stillbirth. Among women with HbA1c levels > 6.6%, every 0.1% increase in the HbA1c level during the perinatal period increased the probability of stillbirth by 2% [44].

The incidence of stillbirth in the present study was 0.56%. The incidence of stillbirth was significantly lower in the good glycemic control group than in the poor glycemic control, while it did not differ among the GDM A1, GDM A2, and PGDM groups. Actively controlling the perinatal blood glucose levels and the maternal-fetal weight gain to remain within the standard target values is an effective way to prevent stillbirth in DIP.

#### LGA

Landon et al. [45] found that insulin treatment decreased the risk of macrosomia from 14.3 to 5.9% among women with GDM. Mackin et al. [27] found that the incidence of macrosomia in T1DM and T2DM was 51 and 38%, respectively. In the present study, the incidence of LGA (all cases of BW > 4000 g) was 3.95%, which is significantly lower than that reported in the literature. This may be related to our team’s guidance on individualized GDM education for DIP patients, the timely guidance for the pregnant women via the new media platform, and the timely adjustment of the diet and exercise plan, which helped to effectively reduce the incidence.

#### Congenital anomalies

Wu et al. [26] found that the aORs of PGDM and GDM for fetal malformations were 2.44 (95% CI: 2.33–2.55) and 1.28 (95% CI: 1.24–1.31), respectively. Nielsen et al. [11] found that increased HbA1c level was related to pregnancy outcomes. For every 1% increase in the HbA1c level, the risk of adverse pregnancy outcomes increased by 3.8–7.3%.

In our study, the incidence of congenital anomalies was 9.37% in the DIP group, and this incidence did not significantly differ among the GDM A1, GDM A2, and PGDM groups, or between the good and poor glycemic control groups.

#### Neonatal asphyxia and NICU transfer

Hyperglycemia and hyperinsulinemia in DIP can affect the biosynthesis of fetal type 2 alveolar cell surface-active substances and the development and maturation of the fetal lungs, which increases the risk of neonatal respiratory distress syndrome, neonatal asphyxia, and transfer to NICU. In the present study, the incidence of neonatal asphyxia was 7.90%. The incidence rates of transfer to the NICU and neonatal asphyxia significantly differed between the GDM A1, GDM A2, and PGDM groups, but did not significantly differ between the good and poor glycemic control groups nor between the insulin and non-insulin groups.

The high incidence of transfer to the NICU in the PGDM group may be related to the high incidence of premature delivery. Clinicians must pay careful attention to prevent premature delivery in women with DIP. For women who are at risk of premature delivery, steroids should be administered in time to promote fetal lung maturation after controlling the blood glucose level to within the standard range. Studies have found that after the administration of steroids to women with DIP, the fasting and postprandial blood glucose levels are increased, and most women need more than twice the previous insulin dose [46]. Therefore, during steroid administration, it is necessary to closely monitor the blood glucose level and to use insulin in a timely manner to reduce the incidence of adverse pregnancy outcomes. Our team found that among women who receive intramuscular injections of dexamethasone 6 mg twice a day for a total of 2 days to promote fetal lung maturation, dexamethasone-induced hyperglycemia can be adequately managed with the concurrent subcutaneous injections of 4–6 U insulin (Detemir) once per day for a total of 2 days.

### Gestational age and mode of delivery

The ACOG guidelines [47] recommend that GDM A1 patients with good glycemic control through exercise and diet and no other indications for induction of labor are not usually recommended to undergo delivery before 39 weeks. In such patients, close monitoring until 40^+ 6^ weeks is appropriate. In the case of GDM A2, patients who need drugs for glycemic control are recommended to deliver at 39 to 39^+ 6^ weeks; for women with poor glycemic control, early delivery is recommended. Delivery at 37–38^+ 6^ weeks can be considered, if the blood glucose level is not well controlled after hospitalization; if the prenatal fetal monitoring is abnormal, delivery at 34–36^+ 6^ weeks should be considered [47]. According to the guidelines for DIP in China, pregnant women with PGDM on insulin therapy can deliver after 39 weeks of gestation if their blood glucose is well controlled, and there are no maternal and fetal complications. If blood glucose control is not satisfactory, or if maternal or fetal complications occur, these patients should be promptly admitted to a hospital for observation, and the timing of delivery should be determined according to their specific condition [21].

### Limitations and future prospects

This was a single-center study, and the number of cases in some subgroups was limited. This study only analyzed the short-term adverse pregnancy outcomes of DIP in mothers and infants. A follow-up study on the long-term effects is ongoing. A multi-center, large-scale, prospective group-controlled study is feasible in the future, as well as long-term follow-up of mothers with DIP and their offspring after delivery.

To determine the increase in the incidence of DIP as compared to the pre-2016 level, the medical history of the patients, including the presence of polycystic ovary syndrome, and their family history should be considered. More details should be collected in future for more in-depth studies.

The incidence of spontaneous premature labor and delivery vs. induction of labor was not evaluated, as the related data were not collected in detail.

The novelty of this study is that it investigated the epidemiological characteristics of DIP, and the outcomes of the second-child policy in terms of DIP, in a treatment center for severely ill pregnant women. The findings of this study may help to provide guidance for standardizing the clinical management of DIP in the future.

## Conclusions

The incidence of DIP has been increasing yearly during the past 5 years. Women with DIP have a high incidence of adverse pregnancy outcomes. Women with high-risk factors for GDM should be screened for diabetes and offered early intervention. However, if only those with high-risk factors for GDM are screened, the missed diagnosis rate will be high. The mean gestational age at the time of the peak insulin dose in women with DIP was 32 weeks. The peak insulin doses for women with PGDM and GDM were 3.67 times and 2 times the initial dose, respectively. In women with DIP, the postpartum insulin dose needs to be reduced to 26–32% of the antepartum dose. Age, weight gain during pregnancy, classification and grading of diabetes, poor IVF-ET, and poor glycemic control are factors that increase the risk of adverse pregnancy outcomes in women with DIP.

## Data Availability

The datasets used and/or analysed during the current study are available from the corresponding author on reasonable request.

## Abbreviations

DIP: Diabetes in Pregnancy
GDM: Gestational Diabetes Mellitus
PGDM: Pregestational Diabetes Mellitus
T1DM: Type 1 Diabetes Mellitus
T2DM: Type 2 Diabetes Mellitus
BMI: Body Mass Index
ART: Assisted Reproductive Technology
IVF-ET: Invitro Fertilization and Embryo Transfer
OGTT: Oral Glucose Tolerance Test
FPG: Fasting Plasma Glucose
HbA1c: Glycohemoglobin
HDP: Hypertensive Disorders of Pregnancy
ICP: Intrahepatic cholestasis of pregnancy
NICU: Neonatal Intensive Care Unit
PROM: Premature Rupture of Membrane
SGA: Small for Gestation Age
LGA: Large for Gestation Age
ACOG: American College of Obstetricians and Gynecologists
PPH: Postpartum Hemorrhage
